# Novel biphasic mechanism of the canonical Wnt signalling component PYGO2 promotes cardiomyocyte differentiation from hUC-MSCs

**DOI:** 10.1007/s00441-023-03774-6

**Published:** 2023-05-26

**Authors:** Yan Shi, Bin Qin, Xiongwei Fan, Yongqing Li, Yuequn Wang, Wuzhou Yuan, Zhigang Jiang, Ping Zhu, Jimei Chen, Yu Chen, Fang Li, Yongqi Wan, Xiushan Wu, Jian Zhuang

**Affiliations:** 1 Guangdong Cardiovascular Institute, Guangdong Provincial People’s Hospital, Guangdong Academy of Medical Sciences, Guangzhou, Guangdong 510100 China; 2 The Center for Heart Development, State Key Laboratory of Development Biology of Freshwater Fish, College of Life Sciences, Hunan Normal University, Hunan Changsha, 410081 China; 3 Laboratory of Artificial Intelligence and 3D Technologies for Cardiovascular Diseases, Guangdong Provincial Key Laboratory of South China Structural Heart Disease, Guangdong Provincial People’s Hospital, Guangdong Academy of Medical Sciences, Guangzhou, 510080 People’s Republic of China; 4 Guangdong Provincial Key Laboratory of Pathogenesis, Targeted Prevention and Treatment of Heart Disease, Guangzhou, Guangdong 510080 People’s Republic of China

**Keywords:** PYGO2, Human umbilical cord-derived mesenchymal stem cells, Canonical Wnt signalling pathway, Cardiomyocyte, β-catenin, PI3K-Akt signalling

## Abstract

Human umbilical cord–derived mesenchymal stem cells (hUC-MSCs) are used to regenerate the myocardium during cardiac repair after myocardial infarction. However, the regulatory mechanism underlying their ability to form mesodermal cells and differentiate into cardiomyocytes remains unclear. Here, we established a human-derived MSCs line isolated from healthy umbilical cords and established a cell model of the natural state to examine the differentiation of hUC-MSCs into cardiomyocytes. Quantitative RT-PCR, western blotting, immunofluorescence, flow cytometry, RNA Seq, and inhibitors of canonical Wnt signalling were used to detect the germ-layer markers *T* and *MIXL1*; the markers of cardiac progenitor cells *MESP1*, *GATA4*, and *NKX2.5* and the cardiomyocyte-marker cTnT to identify the molecular mechanism associated with PYGO2, a key component of the canonical Wnt signalling pathway that regulates the formation of cardiomyocyte-like cells. We demonstrated that PYGO2 promotes the formation of mesodermal-like cells and their differentiation into cardiomyocytes through the hUC-MSC-dependent canonical Wnt signalling by promoting the early-stage entry of β-catenin into the nucleus. Surprisingly, PYGO2 did not alter the expression of the canonical-Wnt, NOTCH, or BMP signalling pathways during the middle–late stages. In contrast, PI3K-Akt signalling promoted hUC-MSCs formation and their differentiation into cardiomyocyte-like cells. To the best of our knowledge, this is the first study to demonstrate that PYGO2 uses a biphasic mechanism to promote cardiomyocyte formation from hUC-MSCs.

**Supplementary Information:**

The online version contains supplementary material available at 10.1007/s00441-023-03774-6.

## Introduction

Cardiovascular disease is a major global health problem (Virani et al. 2020). For example, the American Heart Association reported that cardiovascular disease–related morbidity and mortality are expected to rise to 23.6 million by 2030 (Benjamin et al. 2017). A major concern is the repair and regeneration of the injured heart to address crisis (Benjamin et al. 2017). For this purpose, diverse pluripotent mesenchymal stem cells (MSCs) are commonly used in research, particularly for cardiac regeneration and repair. These cells are most frequently acquired from adipose tissue, bone marrow, and the umbilical cord (Gupta et al. 2021).

Human umbilical cord–derived MSCs (hUC-MSCs) are ideal candidates to observe and understand cardiomyocyte differentiation and potential transplantation (Govarthanan et al. 2020). hUC-MSCs are readily available, can be collected through non-invasive techniques, and are easy to stored and transported (Colicchia et al. 2019; Nagamura-Inoue and He 2014). Furthermore, these pluripotent stem cells can rapidly proliferate, modulate immune responses, express unique combinations of prenatal and postnatal cell phenotypes, and exhibit minimal tumorgenicity and high genomic stability; moreover, they are associated with minimal ethical issues (Colicchia et al. 2019; Nagamura-Inoue and He 2014). Moreover, hUC-MSCs spontaneously express core markers of undifferentiated human embryonic stem cells and early-stage cardiac transcription factors. Additionally, in animal models, hUC-MSCs can be directly differentiated into cardiomyocytes (Colicchia et al. 2019; Santos Nascimento et al. 2014; Wu et al. 2007).

Improved understanding of the biological properties of hUC-MSCs has enables their use in the treatment of heart diseases such as myocardial infraction (MI), chronic ischemic cardiomyopathy (CICM), dilated cardiomyopathy (DCM), and acute myocarditis, as demonstrated in preclinical animal studies (Colicchia et al. 2019; Nagamura-Inoue and He 2014). For example, hUC-MSCs directly transplanted into the MI area in swine model, either intravenously or by coronary injection, can differentiate into cardiomyocytes exhibiting reduced apoptosis and fibrosis in the MI area, as well as enhanced the viable myocardium and improved ventricular remodelling and function (Gao et al. 2015; Martinez et al. 2013; Zhang et al. 2013). Furthermore, hUC-MSCs used to treat DCM can protect the ultrastructure of cardiomyocytes by reducing mitochondrial swelling and maintaining sarcomere integrity (Mao et al. 2017). Moreover, 3 weeks after induced myocarditis, intravenous hUC-MSCs injection reduced inflammatory cell infiltration, adverse cardiac remodelling, and cardiomyocyte apoptosis (Zhang et al. 2017).

An on-going clinical dilemma is that injected cells exhibit a short residence time and that they are removed from the circulation before they can exert a significant therapeutic effect. Therefore, identifying the mechanism underlying hUC-MSCs differentiation into cardiomyocytes will aid in the formation of new novel treatment strategies for cardiomyopathy. Thus, hUC-MSCs have been the focus of numerous investigations on cardiac regenerative therapy (Colicchia et al. 2019). Most methods that use hUC-MSCs typically use transgenic models as well as biological or chemical factors to modulate MSCs differentiation into the cardiac lineage (Ramesh et al. 2021). For example, the addition of 5-Aza, a demethylating agent, to a foetal heart extract promotes the differentiation of hUC-MSCs into cardiomyocytes exhibiting increased expression of sarcomere *alpha-actin* as well as cardiomyogenic markers early-stage (*GATA4*), late-stage (*cTnI*), and mature-stage (*MHC*) cardiac-specific marker *Cx43* (Joshi et al. 2018; Pham et al. 2016). Furthermore, *NKX2.5* overexpression promotes the differentiation of hUC-MSCs into cardiomyocyte-like cells (Ruan et al. 2016). Despite these discoveries, the exact molecular mechanism that regulates the differentiation of hUC-MSCs into cardiomyocytes remains unclear.

PYGO2, one of two members of the pygopus family in vertebrates, was first identified as a core component of the canonical Wnt signalling pathway where it interacts with β-catenin to activate the transcription of its target genes (Belenkaya et al. 2002; Chen et al. 2010; Li et al. 2004). PYGO2 is required for numerous canonical Wnt signalling-dependent organs to function, including in cardiac development. During cardiac (Cantù et al. 2018; Schwab et al. 2007), kidney (Schwab et al. 2007), breast (Schwab et al. 2007), and pancreas (Jonckheere et al. 2008) development, PYGO2 regulates the canonical Wnt signalling mediated development. In contrast, during eye (Song et al. 2007), tooth (Cantù et al. 2017), testis (Cantù et al. 2013; Nair et al. 2008), and salivary gland (de la Roche and Bienz 2007) development, *Pygo2* knockdown does not affect canonical Wnt signalling or disrupt the interaction between Pygo and β-catenin and does not phenocopy the defects of the PYGO2 knockout model. Therefore, PYGO2 regulates the target gene expression dependent or independent of the canonical Wnt signalling.

Animal models have various regulatory mechanisms that control heart development. For example, the interactions between PYGO2 and β-catenin in zebrafish and mice phenocopy the knockout of *Pygo2*, leading to abnormal development of cardiac structure (Cantù et al. 2018); this indicates that Pygo2 signalling depends on canonical Wnt signalling to regulate cardiac development. In a *Drosophila* model, pygo knockdown was found to contribute to the abnormal development of cardiac valves and myofibrils, whereas knockdown of the transcription factors, *Arm/β-Cat*, *lgs/BCL9*, or *pan/TCF*, which mediate canonical Wnt signalling, had little effect on the development of valves and myofibrils (Tang et al. 2014, 2013); this indicates that *pygo* regulates cardiac development independent of canonical Wnt signalling. Furthermore, *PYGO2* promotes the differentiation of hUC-MSCs into cardiomyocyte-like cells (Yang et al. 2020); however, the mechanism through which this occurs dependent or independent of canonical Wnt signalling remains unknown.

To better understand the molecular mechanism through which *PYGO2* regulates hUC-MSCs differentiation into cardiomyocytes, we used a model that faithfully represents the natural phenotype of hUC-MSCs to induce their differentiation into cardiomyocyte-like cells. We demonstrated that *PYGO2* induces hUC-MSCs to form mesodermal-like cells by promoting β-catenin translocation into the nucleus to mediate canonical Wnt signalling during the early stage of differentiation, whereas *PYGO2* induces hUC-MSCs differentiation into cardiomyocytes through the PI3K-Akt signalling during the middle and late stages.

## Materials and methods

### Isolation of hUC-MSCs

hUC-MSCs were isolated from umbilical cords obtained from healthy infants delivered by caesarean section at the Third Xiangya Hospital of Central South University in December 2019. The Ethics Committee of the hospital approved this study (No: 20042), and informed consent was obtained from the mothers. hUC-MSCs were isolated using the following simple method (Ruan et al. 2016; Yang et al. 2020): blood was washed off the cord using PBS (Hyclone) and the umbilical artery and umbilical vein were peeled off using forceps. The cord was then cut into small pieces (3–5 mm^3^) and evenly distributed in a 10-cm culture dish (Corning). Next, 5 mL of serum-free DMEM/F12 (Hyclone) medium was added so that the tissue block was immersed shallowly instead of being suspended in the medium. Finally, the cells were incubated at 37 °C in an incubator (Thermo Fisher Scientific) containing 5% CO_2_. After 6 h, DMEM/F12 medium containing 10% FBS (TransGen Biotech) was added, and the culture was continued for 2 days; the medium was refreshed with fresh medium containing 10% FBS.

After 7 days, a small number of spindle cells began to traverse the tissue block (Supplementary Fig. S1a) and a larger number of spindle cells were observed on day 14 (Supplementary Fig. S1b). Cells were suspended in 0.0125 g/mL trypsin and then passaged at 80–90% confluence. Cells remained spindle-shaped and rapidly proliferated with further passage (Supplementary Fig. S1c, d).

### Culture and identification of hUC-MSCs

Cells at passage 3 were identified for stemness by flow cytometry using antibodies against the following CD markers (purchased from BD Biosciences): CD11b-PE, CD29-PE, CD31-PE, CD34-PE, CD73-PE, CD90- PE, HLA-DR-PE, CD105-APC, and CD45-FITC. The cells were incubated with nonspecific IgG as a control. Cells were induced to undergo adipogenic or osteogenic differentiation using a special induction medium (FY200007 and FY200006, Fuyuan Bio) based on the manufacturer’s instructions. The general method included adding cells (1–2 × 10^5^ cells/per well) to 6-well plates, followed by the addition of special induction medium 24 h later, with a medium change every 3 days. After 21 days, the cells were stained with Oil Red O staining or Alizarin Red staining, which indicates the adipogenic and osteogenic differentiation abilities of hUC-MSCs, respectively.

### Lentivirus infection of hUC-MSCs

The human gene encoding PYGO2 (NM_138300.4) was ligated into the lentivirus vector (GV492) encoding puromycin resistance and green fluorescent protein (GFP). The empty vector was used as a control and designated as GV492-Vector. The PYGO2 overexpression vector was designated GV492-PYGO2. For PYGO2 knockdown, three PYGO2-specific siRNA sequence 5′‑ccggTACTCACATCTGACGGAGTTTctcgagAAACTCCGTCAGATGTGAGTAtttttg‑3′, 5′‑ccggCCTTCTCTGTCCCAACGATTTctcgagAAATCGTTGGGACAGAGAAGGtttttg‑3′, 5′‑ccggTGTCGGAGTGAGGTGAACGATctcgagATCGTTCACCTCACTCCGACAtttttg‑3′ (si-PYGO2-1, si-PYGO2-2, and si-PYGO2-3, respectively) were ligated into the GV493 lentivirus vector containing the puromycin resistance and green fluorescent protein (GFP) genes. The GV493 empty vector served as a control and was labelled as si-CT. All recombinant lentivirus vectors and the empty vector were packaged using an infection system acquired from Shanghai Genechem. Fourth passage hUC-MSCs were added to a 100-cm culture dish and, upon reaching 70–80% confluence, incubated with the GV492-PYGO2 (MOI = 50) and GV492-Vector (MOI = 50), and designated PYGO2 and Vector, respectively. Fourth passage hUC-MSCs were also incubated with si-CT, si-PYGO2-1, si-PYGO2-2, and si-PYGO2-3 when they reached 70–80% confluence. After 72 h, the uninfected cells were eliminated using 2 µg/mL puromycin (Sangon Biotech); when the percentage of GFP-positive cells was > 90%, the infection was considered successful.

### RNA isolation and qRT-PCR

Collection cells then homogenized the cells in TRIzol (Life) and extracted with chloroform isoamyl alcohol (Shi et al. 2020a). The total RNA was synthesized into cDNA according to the instructions (Trans, TransScript One-Step gDNA Removal and cDNA Synthesis SuperMix), and qRT-PCR was performed using standard PCR conditions using an Applied Biosystems Quanstudio 5 machine with SYBR Green PCR Master Mix (Takara). The gene expression levels were standardized to GAPDH expression. All the data were analysed using the 2-ΔΔCT Livak method, and *p* < 0.05 is considered significant. The data are presented in the form of a histogram that were generated by GraphPad Prism 8.0.2. All primers are shown in Supplementary Table S1.

### Western blotting

Total protein samples were prepared in radioimmunoprecipitation assay (RIPA) buffer; then, the protein concentration was determined by BCA assay (Beyotime). The total protein were separated by electrophoresis through Future PAGETM 4–12% (ACE, 11 Wells), then transferred the protein to PVDF membranes (Millipore, 0.45 µm), blocked with 8% skim milk and incubated with anti-PYGO2 (1:1000, Genetex), anti-NXK2.5 (1:1000; Invitrogen), anti-GATA4 (1:1000, Proteintech), anti-cTnT (1:1000, Abcam), anti-β-catenin (1:1000, Proteintech), anti-β-Tubulin (1:5000, ABcanol), anti-ACTA2 (1:1000, Abcam), anti-GAPDH (1:5000, ABcanol), and anti-β-ACTIN antibody (1:5000 dilution, ABcanol). The signal densities of the target protein bands were quantified and normalized to β-ACTIN or GAPDH using Image J. Nucleo-cytoplasmic isolation was performed according to the instructions of the Nuclear and Cytoplasmic Protein Extraction Kit (Sangon Biotech), followed by routine protein isolation and antibody incubation. As for the analysis of nuclear localization of β-catenin, we used the formula [(β-catenin/H3)-(Protein/GAPDH) + 1] (in both the vector and PYGO2 groups) to exclude for cytopasmic contamination.

### Immunofluorescence analysis

Immunofluorescence assays were performed as follows: cells (1 × 10^5^ cells/well) were seeded into 12-well plates with a round coverslip (Biosharp) and cultured for 12–24 h. The cells were then fixed with 4% paraformaldehyde (YuanYe Bio-Technology) for 20 min at room temperature and then permeabilized with 0.5% Triton X-100 (Solarbio) for 15 min at room temperature. Nonspecific antibody binding was blocked by washing the cells with 5% normal goat working serum (Solarbio) for 1 h at room temperature, and the cells were incubated with NKX2.5 antibody (1:200, Abcam), GATA4 antibody (1:100, Proteintech), β-catenin (1:500, Proteintech), and cTnT antibody (1:500, Abcam) overnight at 4 ℃. The secondary antibody (Alexa Fluor 568, Invitrogen) was incubated with the cells at room temperature for 1.5–2 h, and DAPI (1:5000, Beyotime Biotechnology) was added for 10 min to stain the nucleus. Images were acquired using a fluorescence microscope (Carl Zeiss).

### Flow cytometry

For flow cytometry, the cells were collected and re-suspended in an appropriate amount of PBS containing 0.1% FBS; the cell density was then adjusted to approximately 5 × 10^6^ cells/mL. The cell suspension (200 µL) was added to a flow tube, incubated in fixation buffer (Biolegend) for 20 min at room temperature in the dark, incubated with 5 µL of PE-labelled monoclonal antibody cTnT (bs-2804R, Bioss), thoroughly mixed and then incubated in the dark for 15 min. The proportion of cell subsets was determined using a flow cytometer (CytoFLex, Beckman).

### Dual-Luciferasle reporter system analysis

GV492-Vector and GV492-PYGO2 infected HEK cell lines, and transfected top flash plasmid and phRL-TK plasmid using Lipofectamine 8000 (Beyotime Biotechnology) according to the instructions. After 24 h, luciferase activity was measured using Dual-Luciferase Reporter Assay System (Promega). The renilla luciferase activity was normalized to the firefly luciferase activity when comparing the wild type and overexpression group.

### RNA-seq

The control and PYGO2 groups (three biological replicates per group) were sequenced used a BGISEQ platform. Before data analysis, low quality reads, reads with adaptor sequences, and reads with high levels of N-bases were filtered. The curated sequences were aligned to a reference genome (GCF_000001405.39_GRCh38.p13) using HISAT and to reference genes using Bowtie2.

### Statistical analysis

Data are presented as the mean and SD. Student’s *t*-test and one-way analysis of variance were used to evaluate comparisons between two and three groups, respectively. A *p*-value of < 0.05 was considered statistically significant (*p < 0.05; **p < 0.01; ***p < 0.001).

## Results

### PYGO2 overexpression induces the differentiation of hUC-MSCs into cardiomyocyte-like cells

Isolated undifferentiated hUC-MSCs were analysed through flow cytometry to detect their adipogenic and osteogenic (Oil Red O and Alizarin Red staining, respectively) stem cell potential (Supplementary Fig. S2). The results indicated that hUC-MSCs expressed the CD29, CD73, CD90, CD105, CD34, CD45, and HLA-DR markers but not CD31 or CD11b (Supplementary Fig. S2a-i). Further, Oil Red O and Alizarin Red staining indicated that they exhibited adipogenic and osteogenic potential (Supplementary Fig. S2j, k). These results are consistent with that of a previous report (Ruan et al. 2016).

**Fig. 1:**
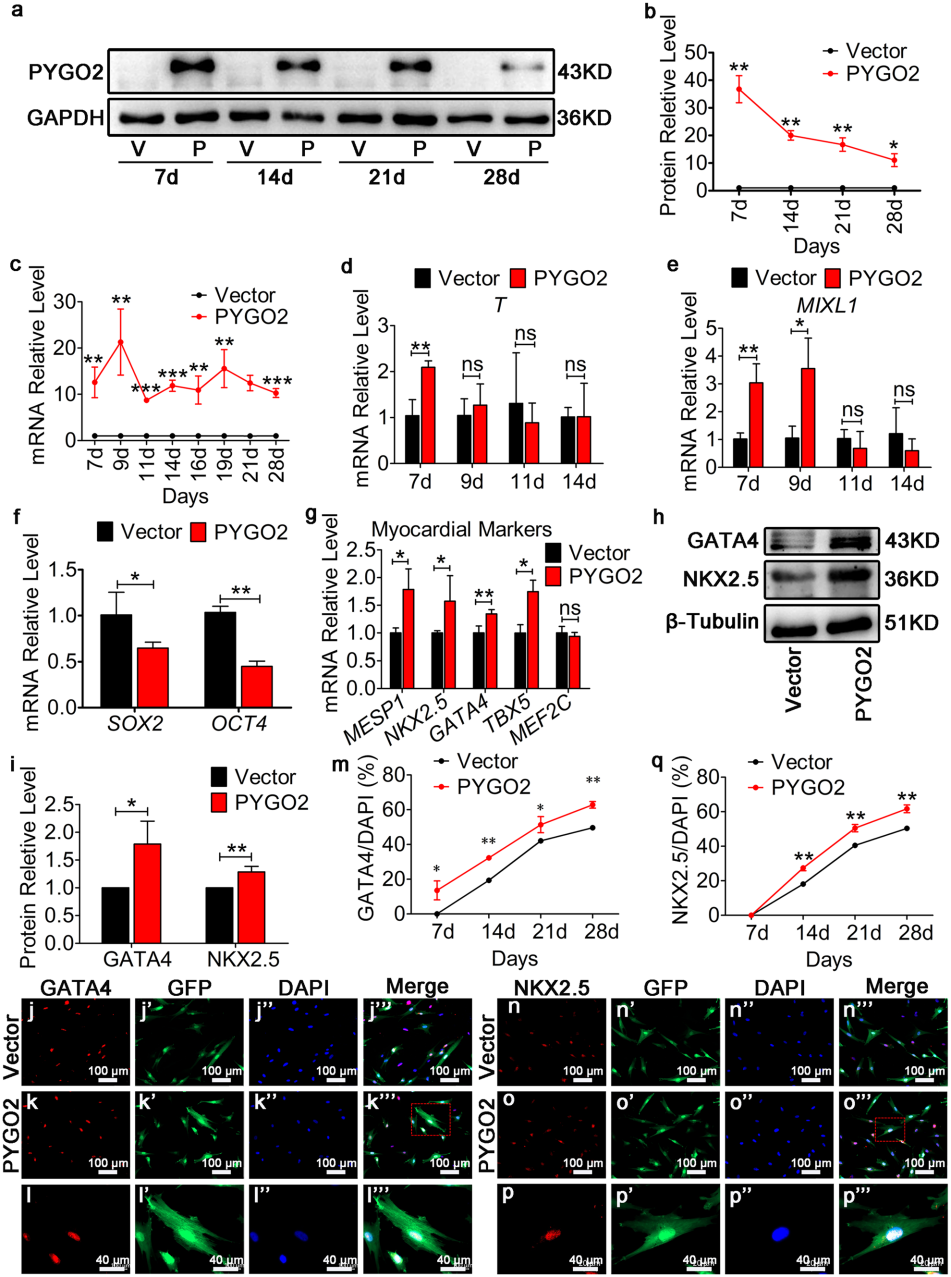
PYGO2 promotes the expression of myocardial mesodermal genes in hUC-MSCs. (**a**) Expression of PYGO2 on different days determined through western blotting analysis. V, vector, the group infected with empty vector, used as control; P, PYGO2, the group that overexpressed PYGO2. (**b**) Quantitative plot of a. (**c**) Expression of PYGO2 on different days measured using qRT-PCR. (**d **and **e**) qRT-PCR of the expression of *T* (*Brachyury*) and *MIXL1* on different days. (**f**) Expression of *OCT4* and *SOX2* measured using qRT-PCR when PYGO2 was overexpressed on day 7. (**g**) qRT-PCR detection of the expression of myocardial markers on day 9. (**h**) Western blotting analysis of the expression of NKX2.5 and GATA4 on day 9. (**i**) Quantitative plot of h. (**j**–**q**) Immunofluorescence detection of NKX2.5- and GATA4-positive cells. (**l**–**p**) The enlarged images shown in the red boxes of k‴ and o‴, respectively. (**m**–**q**) The proportions of GATA4- and NKX2.5-positive cells at each stage, respectively. Vector, the group infected with empty vector, was used as control; PYGO2, the group that overexpressed PYGO2. d, days; *p < 0.05; **p < 0.01; ***p < 0.01. Error bars represent the mean and SD

Fourth passage hUC-MSCs were infected with a lentivirus vector (day 0), and uninfected cells were eliminated by puromycin treatment on days 3–6. On day 6, 95% of the cells expressed GFP (Supplementary Fig. S3); thus, a PYGO2 stable line was established. PYGO2 expression was observed in the hUC-MSC-PYGO2-overexpressing group (PYGO2) and the hUC-MSC-Vector control group (Vector) using qRT-PCR (days 7, 9, 11, 14, 16, 19, 21, and 28) and western blotting analysis (days 7, 14, 21 and 28). These results indicate that the protein and mRNA expression in the PYGO2 groups was significantly and highly expressed at all time-points (Fig. 1(a–c)).

Stem cells may be induced to form a mesodermal-like cell (Protze et al. 2019). Therefore, we determined the effects of PYGO2 on the differentiation of hUC-MSCs into mesodermal-like cells. We detected the expression of the mesoderm markers *T* (*Brachyury*) and *MIXL1* on day 7 (second day after establishing the stable PYGO2-expressing cell line) as well as on days 9, 11, and 14. qRT-PCR results indicated that the levels of *T* and *MIXL1* mRNAs were significantly upregulated on day 7 (Fig. 1(d, e)); however, there were no differences on days 9, 11, or 14 other than the fact that *MIXL1* was upregulated on day 9 (Fig. 1(d, e)). These results indicate that PYGO2 overexpression induced hUC-MSCs to form mesodermal-like cells on day 7. Concurrently, *PYGO2* overexpression was significantly associated with decreased expression of the stem cell markers *OCT4* and *SOX2* on day 7 (Fig. 1(f)). Thus, *PYGO2* overexpression induces hUC-MSCs to form mesodermal-like cells by day 7.

We measured the levels of the cardiac progenitor cell marker *MESP1* and the key cardiac development transcription factors *GATA4*, *NKX2.5*, *TBX5*, and *MEF2C* on day 9. The qRT-PCR results revealed that the levels of all but *MEF2C* (unchanged) were significantly upregulated (Fig. 1(g)). Western blotting analysis revealed elevated levels of GATA4 and NKX2.5 on day 9 (Fig. 1(h, i)). Further, immunofluorescence analysis indicated that the proportion of GATA4-positive cells increased on day 7 in the PYGO2 group, whereas the proportion of GATA4-positive cells in the PYGO2 group was significantly higher compared with that in the control group (Fig. 1(j–m); Supplementary Fig. S4). The number of NKX2.5-expressing cells was significantly higher on day 14 than in the Vector group during the subsequent analyses (Fig. 1(n–q); Supplementary Fig. S4). These results indicate that PYGO2 overexpression is associated with the differentiation of hUC-MSCs into mesodermal-like cells on day 7 and the further differentiation into cardiac progenitor cells by day 9.

**Fig. 2:**
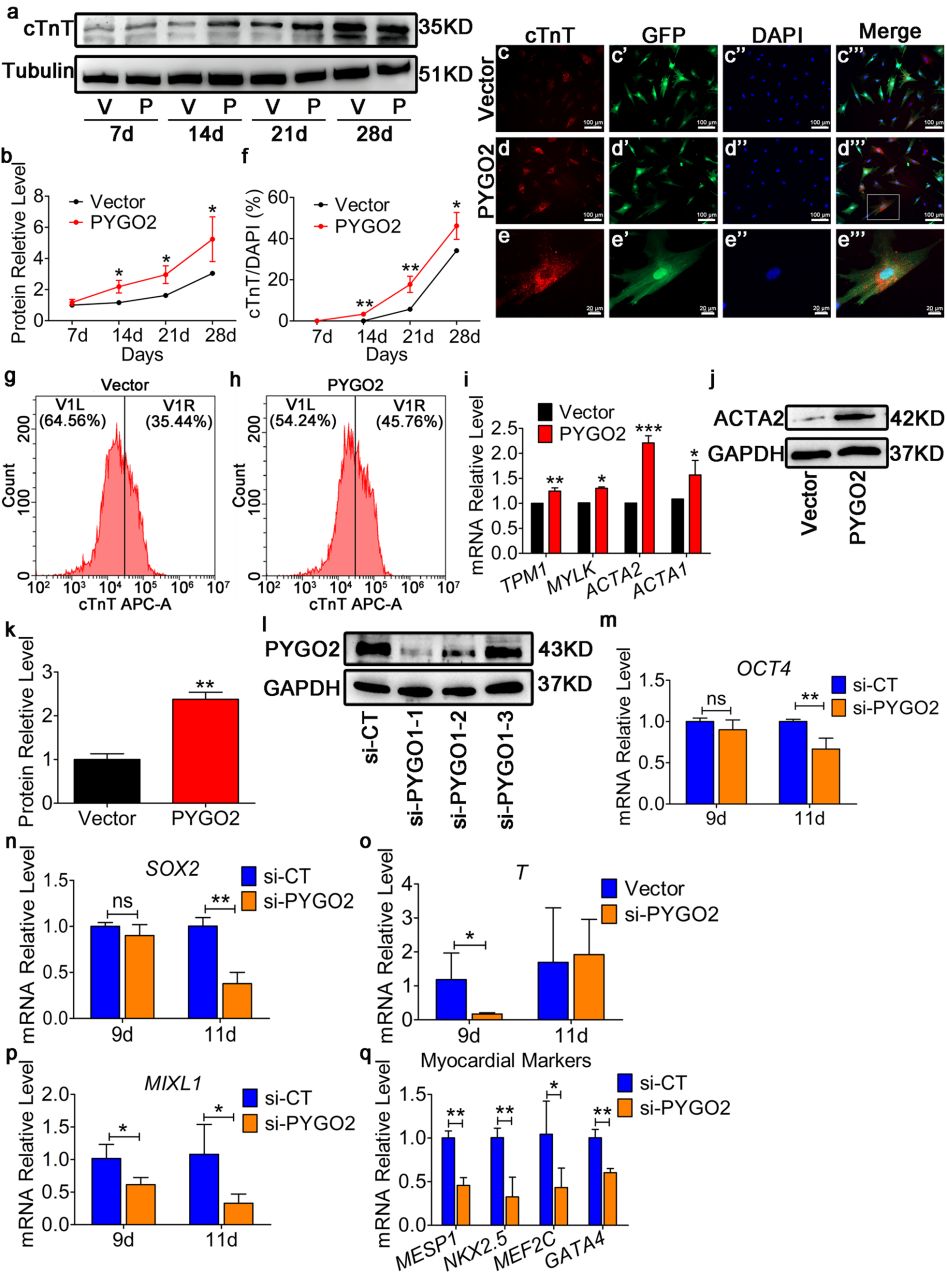
Overexpression PYGO2 promotes the differentiation of hUC-MSCs into cardiomyocytes. (**a**) Western blotting analysis showing the temporal expression of the cardiomyocyte marker factor cTnT. (**b**) Quantitative plot of a. (**c**–**d**) The cardiomyocyte-marker cTnT was detected using immunofluorescence on day 28. (**e**) The enlarged images shown in the white boxes of d‴. (**f**) Statistical analysis of **c**–**d**. The percentage of cTnT-positive cells (cTnT/DAPI) on different days. (**g**–**h**) Number of cTnT-positive cells among the total cardiomyocytes detected using flow cytometry on day 28. (**i**) qRT-PCR of the expression of the cardiomyocyte-markers, *TPM1*, *MYLK*, *ACTA1*, and *ACTA2* on day 28. (**j**) Western blotting analysis of the expression of cardiomyocyte marker factor ACTA2 on day 28. (**k**) Quantitative plot of j. (**l**) Western blotting analysis of the efficiency of PYGO2 knockdown on day 7. (**m**–**n**) qRT-PCR of expression of *OCT4* and *SOX2* on days 9 and 11 after PYGO2 knockdown. (**o**–**p**) qRT-PCR of the expression of mesodermal markers *T* and *MIXL1*, respectively, on days 9 and 11. (**q**) qRT-PCR of the expression of myocardial markers *MESP1*, *NKX2.5*, *MEF2C*, and *GATA4* on day 11 after PYGO2 knockdown. Vector, the group infected with empty vector, served as the control; PYGO2, the group that overexpressed PYGO2; si-CT, the control group of PYGO2 knockdown; si-PYGO2, the group that PYGO2 knockdown. *p < 0.05; **p < 0.01; ***p < 0.001; ns, *p* > 0.05. Error bars indicate the mean and SD; d, days

Next, we found that the expression of the cardiomyocyte-marker factor cTnT was unchanged on day 7, but had significantly increased on days 14, 21, and 28 (Fig. 2(a, b)). Immunofluorescence analysis revealed that PYGO2 overexpression resulted in approximately 3% cTnT-positive cells on day 14 (*p* < 0.01), 10% (*p* < 0.01) on day 21 and 14% on day 28 (*p* < 0.01) compared with the Vector group (Fig. 2(c–f); Supplementary Fig. S4). Over time, the percentage of cTnT-positive cells increased, suggesting that more mesodermal-derived hUC-MSCs entered cardiomyogenic fate following PYGO2 overexpression. These results were confirmed using flow cytometry analysis (Fig. 2(g, h)). Furthermore, the expression of other cardiac markers, including *ACTA1*, *ACTA2*, *TMP1*, and *MYHLK*, increased on day 28 as measured using qRT-PCR (Fig. 2(i)). Western blotting analysis revealed that the ACTA2 protein was also upregulated (Fig. 2(j, k)). The above data indicate that PYGO2 overexpression induces the differentiation of hUC-MSCs into cardiomyocyte-like cells.

To determine the effect of PYOG2 knockdown on hUC-MSCs, siRNA interference lentivirus GV493 was generated to knock-down PYGO2 (si-PYGO2). We designed three siRNA sequences, designated si-PYGO2-1, si-PYGO2-2, and si-PYGO2-3 along with GV493-empty (si-CT) as a control. After 7 days of infection with hUC-MSCs, the knock-down efficiency was assessed by western blotting analysis. The results indicated that si-PYGO2-1 was the most efficient siRNA for downregulating the expression of PYGO2 (Fig. 2(l)); thus, we selected this construct to perform the PYGO2 knock-down experiments. First, we used qRT-PCR to detect the expression of the stem cells markers *OCT4* and *SOX2* on days 9 and 11. The results indicated that *OCT4* and *SOX2* were unchanged on day 9, but were downregulated on day 11 (Fig. 2(m, n)). Next, we measured the expression of the mesodermal markers *T* and *MIXL1* on days 9 and 11; the myocardial mesodermal marker *MESP1*, and the myocardial markers *GATA4*, *NKX2.5*, and *MEF2C* on day 11. The results indicated that they were all downregulated on day 11 (Fig. 2(o–q)). These results indicated that following PYGO2 knock-down, hUC-MSCs differentiate into other cells, instead of cardiomyocytes; however, the specific differentiated cells need to be identified.

### PYGO2 initiates the differentiation of hUC-MSCs into cardiomyocyte-like cells through the canonical Wnt signalling pathway during the early stage

**Fig. 3:**
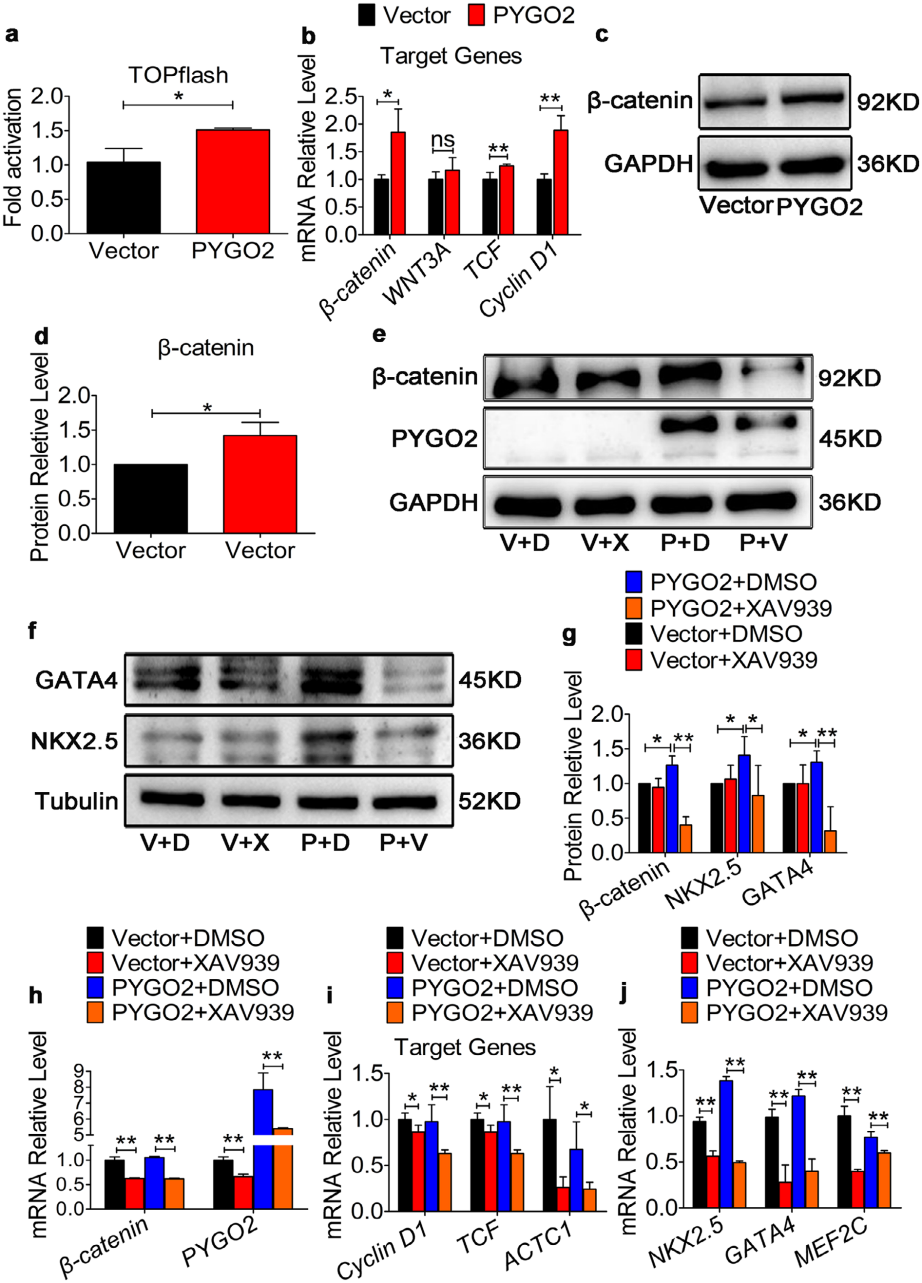
PYGO2-dependent Wnt/β-catenin signalling promotes the differentiation of hUC-MSCs into cardiomyocytes. **a **Dual-luciferase detection of Wnt/β-catenin signalling activity in the HEK cell line. **b **qRT-PCR of the expression of Wnt/β-catenin signalling core members and its target gene on day 7. **c **Western blotting analysis of atmosphere β-catenin expression on day 7. **d **Quantification of β-catenin levels using a greyscale analysis in c. **e**–**f** After adding the Wnt/β-catenin inhibitor, XAV939, the expression of β-catenin, PYGO2, NKX2.5, and GATA4 was determined through western blotting on day 9. V + D, Vector + DMSO: the same dose of inhibitor solvent DMSO was added to the empty group; V + X, Vector + XAV-939: the empty group was added to XAV-939 dissolved in DMSO. P + D, PYGO2 + DMSO: DMSO was added to the PYGO2-overexpression group; P + X, PYGO2 + XAV-939: PYGO2 group was added to XAV-939. **g **Quantification of genes using the greyscale analysis in e and f. **h**–**j** After adding the inhibitor, the expression of β-catenin, PYGO2, and target genes of Wnt/β-catenin signalling (*Cyclin D1*, *TCF*, and *ACTC1*) and myocardial markers (*NKX2.5*, *GATA4*, and *MEF2C*) was measured through qRT-PCR on day 9. Vector, the group infected with empty vector, was used as a control; PYGO2, the group that overexpressed PYGO2; *p < 0.05; **p < 0.01. Error bars represent the mean and SD

We determined whether PYGO2 can induce the differentiation of hUC-MSCs into cardiomyocytes through the canonical Wnt signalling pathway. Stable *PYGO2*-overexpressing HEK cells were established (Supplementary Fig. S5) by transfection with the Topflash plasmid (containing the β-catenin reporter TCF/LEF sites upstream of a luciferase reporter (Veeman et al. 2003)), and the activity of the canonical Wnt signalling pathway was measured. The results indicated that *PYGO2* overexpression increased surrogate β-catenin signalling and slightly enhanced canonical Wnt signalling by approximately 1.5-fold (Vector group = 1.03; PYGO2 group = 1.51; *p* < 0.05) (Fig. 3a). Moreover, qRT-PCR and western blotting analysis revealed that PYGO2 overexpression promoted the expression of β-catenin on day 7 compared with the Vector control (Fig. 3b–d). Furthermore, the expression of the downstream components of canonical Wnt signalling *TCF* and *Cyclin D1* increased on day 7 (Fig. 3b); however, the expression of *Wnt3a*, which encodes the receptor that mediates the initial Wnt signalling event, was unchanged (Fig. 3b). There results indicate that PYGO2 expressed by hUC-MSCs regulates the expression of downstream genes by attenuating the expression of β-catenin.

**Fig. 4:**
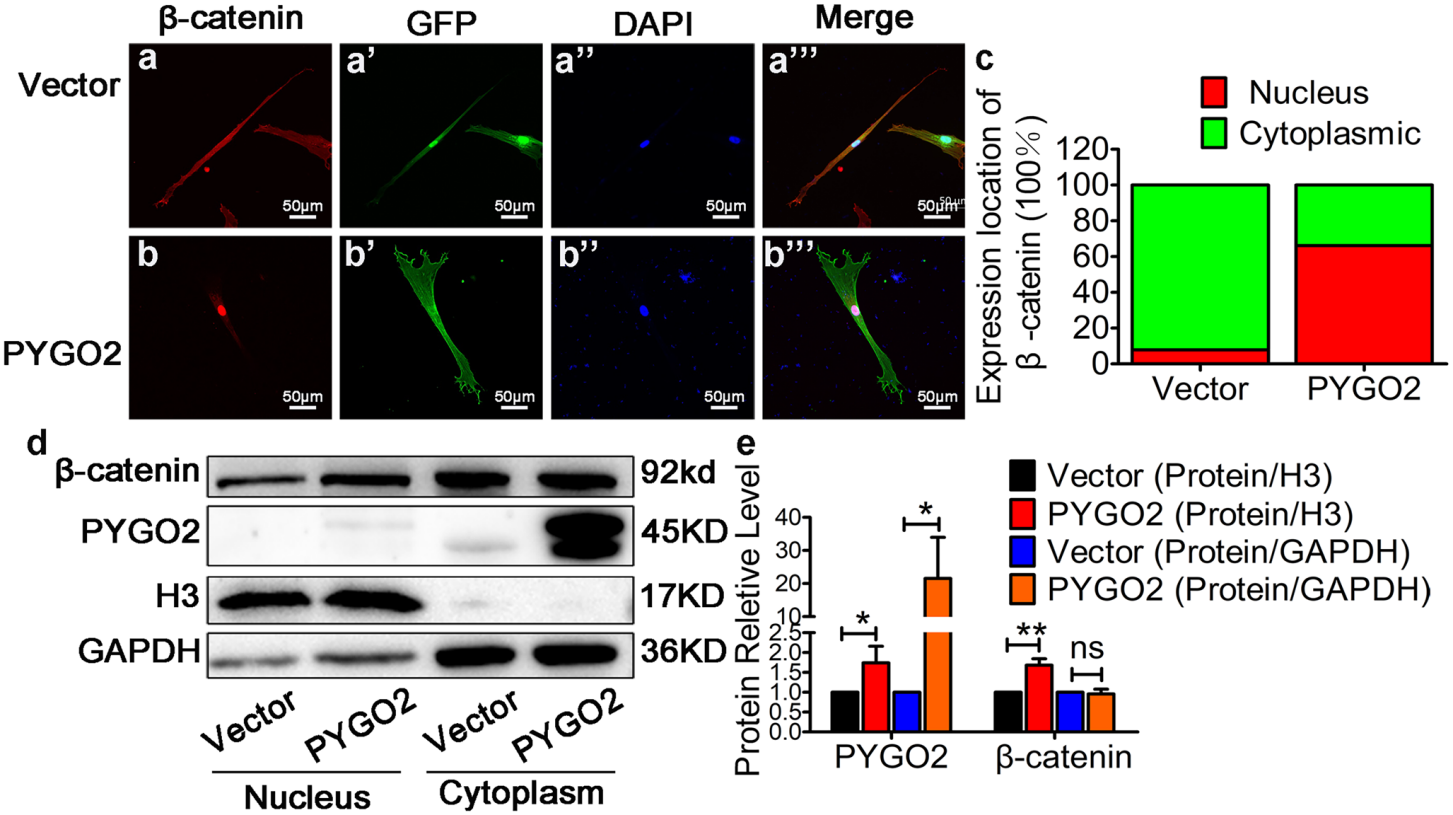
PYGO2 promotes nuclear translocation of β-catenin. (**a–b**) Immunofluorescence indicating the expression of β-catenin on day 7. β-catenin, the expression of β-catenin; GFP, the expression of PYGO2; DAPI, indicating the nucleus; Merge, the overlap of β-catenin, PYGO2 and DAPI. Scale bar, 50 μm. (**c**) Data statistics of a–b. Nuclear, the expression of β-catenin mainly located in the nucleus. Cytoplasmic, the expression of β-catenin mainly located in the cytoplasm. Each group contained at least 100 cells, which expressed both GFP and β-catenin. (**d**) Western blotting analysis of the cytoplasmic and nuclear expression of β-catenin and PYGO2 on day 7. H3, the internal reference antibody for the nucleus; GAPDH, the internal reference antibody for the cytoplasm. (**e**) Quantitative plot of b. Vector, the group infected with empty vector, which was used as a control; PYGO2, the group that overexpressed PYGO2; *p < 0.05; **p < 0.01; ns, *p* > 0.05. Error bars represent the mean and SD

Next, we used XAV939, which selectively inhibits transcription mediated by canonical Wnt signalling and reduces β-catenin expression (Shi et al. 2022), to treat the Vector and PYGO2 groups (DMSO as a control). Following treatment with Vector + DMSO, Vector + XAV939, PYGO2 + DMSO, and PYGO2 + XAV939, we found that XAV939 decreased the expression of β-catenin in the Vector group and PYGO2 group (Fig. 3e, g, h). Furthermore, XAV939 decreased the expression of PYGO2 (Fig. 3e, h), which is consistent with a previous study showing that the expression of PYGO2 is altered by β-catenin expression (Fiedler et al. 2015; Townsley et al. 2004). When XAV939 was used to treat the PYGO2 + XAV939 group, *PYGO2* overexpression decreased the expression of the canonical Wnt signalling target genes *Cyclin D1*, *TCF*, and *ACTC1* and that of the myocardial markers *NKX2.5*, *GATA4*, and *MEF2C* compared with PYGO2 + DMSO (Fig. 3f–j). Although the differences were great, the results were statistically significant and showed that PYGO2 initiates the events leading to the generation of cardiomyocyte-like cells through hUC-MSCs differentiation mediated by the Wnt signalling pathway at an early stage. *pygo* acts downstream or in parallel with Arm to regulate the nuclear function of the Arm protein during embryogenesis and development of the imaginal disc in *flies* (Belenkaya et al. 2002; Shi et al. 2020b). Moreover, in the immortalized monkey kidney-derived COS cell line and the human colorectal cancer-derived SW480 cell line, PYGO2 promotes the nuclear translocation of β-catenin into the nucleus (Townsley et al. 2004). In these cells, the transcriptional complex formed by β-catenin is localized to the chromatin through Pygo (Townsley et al. 2004); however, whether PYGO2 promotes the nuclear translocation of β-catenin in stem cells is unclear. We used immunofluorescence to determine the localization of β-catenin on day 7. The results indicated that in the control group, β-catenin was mainly located in cytoplasm; however, in the PYGO2 group, β-catenin was primarily located in the nucleus (Fig. 4a–c). Subcellular fractionation revealed that the cytoplasmic levels of β-catenin were unchanged, whereas the nuclear expression of β-catenin was upregulated (Fig. 4d, e). These results indicate that in hUC-MSCs, PYGO2 overexpression increases β-catenin levels and increased the level enter nucleus.

### PYGO2 promotes the persistent differentiation of cardiomyocyte-like cells via the PI3K-Akt signalling pathway

**Fig. 5:**
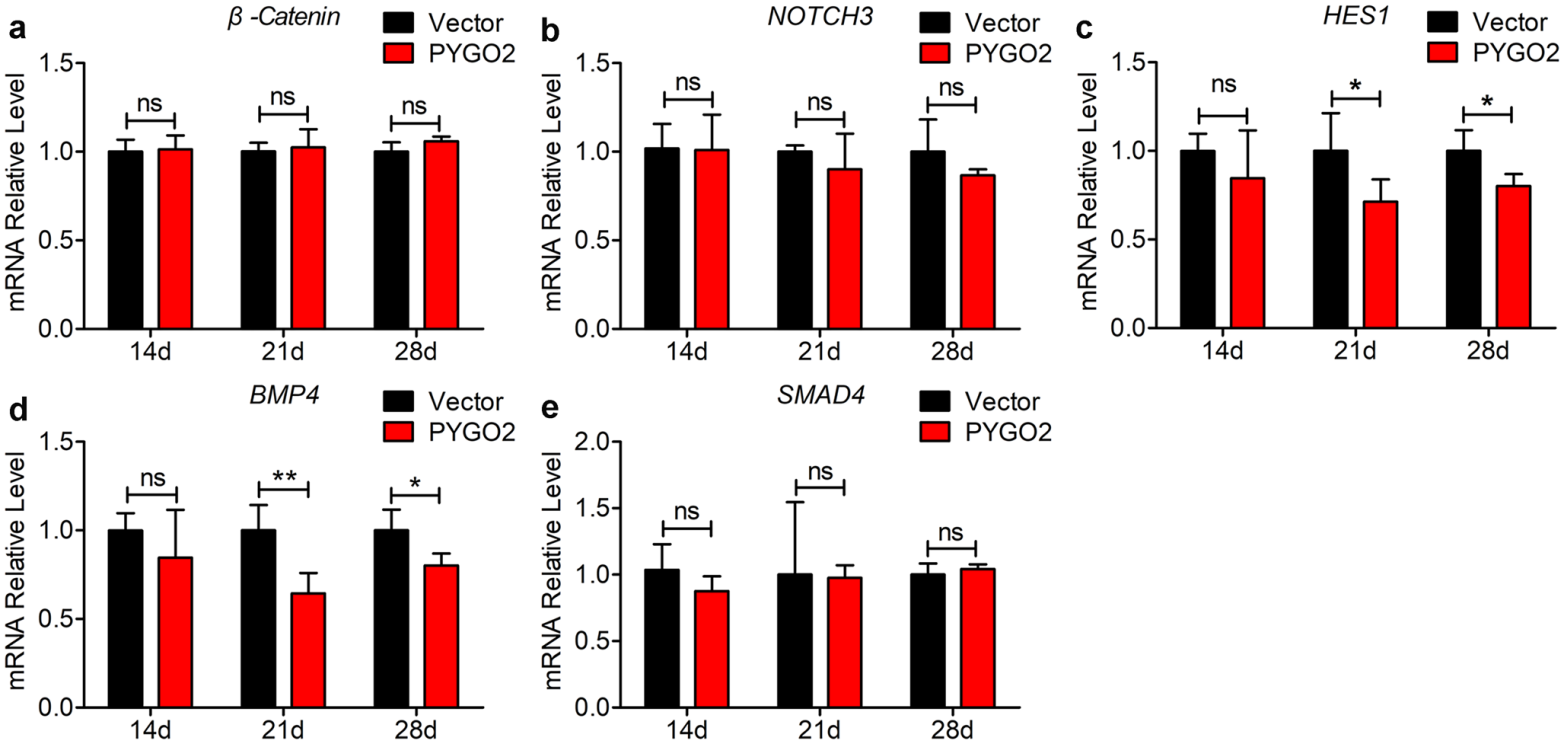
qRT-PCR analyses the expression of NOTCH and BMP signalling pathway components. **a** qRT-PCR of the temporal expression of β-catenin. **b**–**e** qRT-PCR of temporal expression of the NOTCH and BMP signalling pathway components *NOTCH3*, *HES1*, and *BMP4*, *SMAD4*. Vector, the group infected by empty vector (control); PYGO2, the group that overexpressed PYGO2; *p < 0.05; **p < 0.01; ns, *p* > 0.05. Error bars represent shows the mean and SD

Canonical Wnt signalling induces mesodermal cells to differentiate into cardiac progenitor cells, which is inhibited at a later stage, to maintain the development of the ventricular myocardium (Burridge et al. 2015). Here, we did not detect temporal changes during the three stages of differentiation on days 14, 21, and 28 (Fig. 5a). These data support the conclusion that PYGO2 regulates the differentiation of hUC-MSCs into cardiomyocytes independent of canonical Wnt signalling during the middle–late stage.

PYGO2 interacts with histones, and the resulting complexes act as transcriptional co-factors to regulate gene expression (Miller et al. 2013; Shi et al. 2020b). For example, in MaSC/basal cells, PYGO2 represses the chromatin state at the *NOTCH3* locus to inhibit its expression (Gu et al. 2013). NOTCH and canonical Wnt signalling synergistically regulate the expression of *BMP4* and *SMAD4* to promote normal organ development (Lee et al. 2009; Markouli et al. 2021; Szemes et al. 2020). Here, we detected the expression of the NOTCH signalling pathway component *NOTCH3* and the NOTCH target *HES1* as well as *BMP4* and *SMAD4*, which are components of the BMP signalling pathway. The results indicated that *NOTCH3* and *SMAD4* levels were unchanged on days 14, 21, and 28 (Fig. 5b, e). *HES1* and *BMP4* mRNA levels did not change on day 14 but decreased slightly on days 21 and 28 (Fig. 5c, d). These results indicate that PYGO2 does not regulate the differentiation of hUC-MSCs into cardiomyocytes cell through NOTCH and BMP signalling during the middle–late stages.

**Fig. 6:**
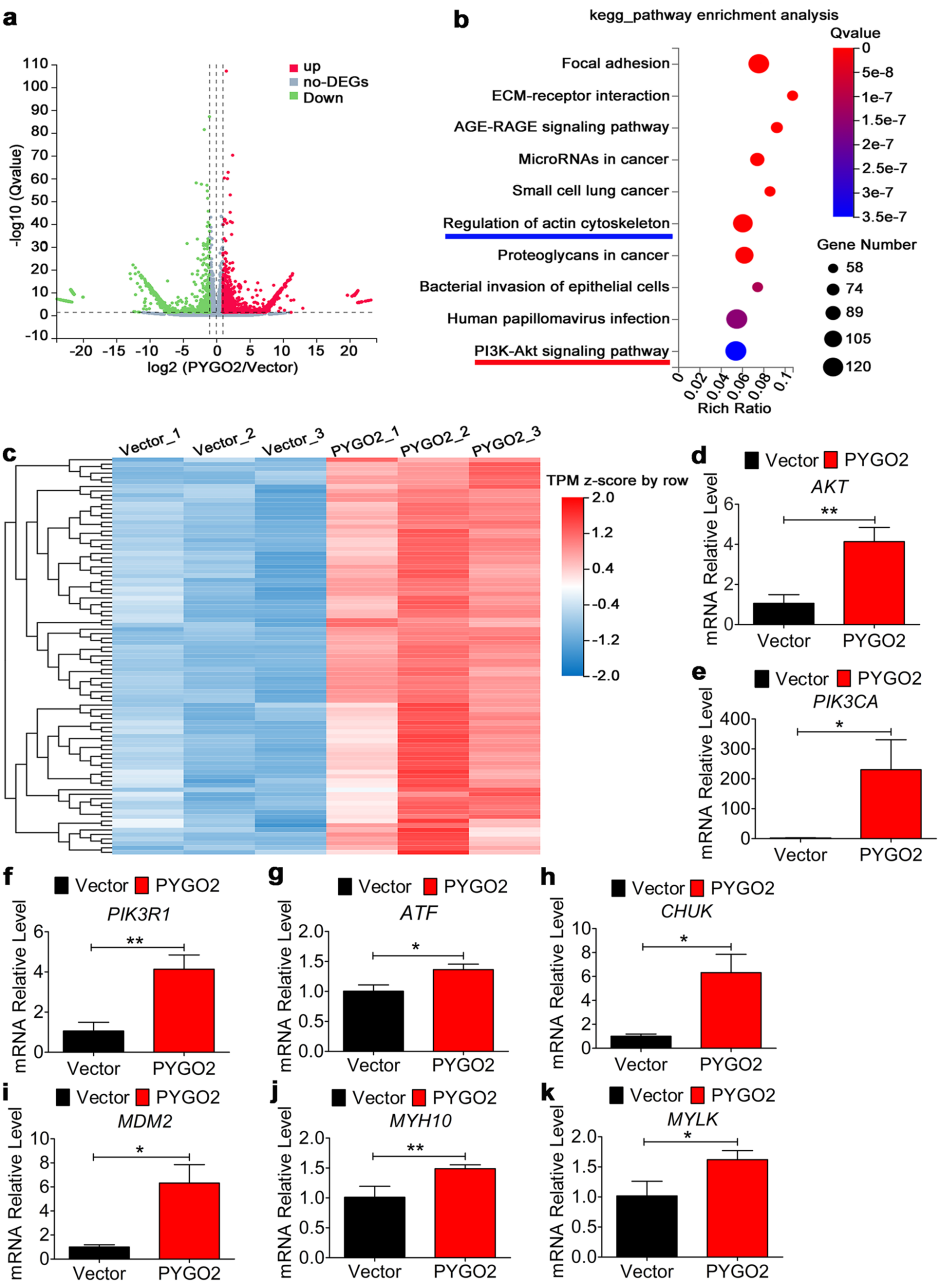
PYGO2 regulates cardiomyocyte differentiation and maturation independent of Wnt/β-catenin signalling. **a **Volcano plot of the data showing changes in expression identified by RNA-seq analysis (PYGO2/Vector). **b **KEGG pathway enrichment analysis of 3114 upregulated genes. **c **Heatmap of the PI3K-Akt signalling pathway. All screened genes are *p* < 0.05. **d**–**i** qRT-PCR of the expression of the members and target genes of the PI3K-Akt signalling pathway. **j**–**k** qRT-PCR detection of the expression of the actin cytoskeleton. Vector, the group infected by empty vector (control); PYGO2, the group that overexpressed PYGO2; *p < 0.05; **p < 0.01; ns, *p* > 0.05. Error bars represent shows the mean and SD

To determine how PYGO2 regulates the generation of cardiomyocytes during the middle–late stages, we screened for differentially expressed genes in the middle–late stage (day 16) and identified 3114 genes that were upregulated and 2832 genes were downregulated in the PYGO2 group compared with the Vector control group (Fig. 6a). KEGG pathway enrichment analysis of the 3114 upregulated genes revealed that they were mainly enriched in the actin cytoskeleton and PI3K-Akt signalling pathway (Fig. 6b, c). Measuring the levels of the PI3K-Akt signalling pathway genes *AKT*, *PIK3CA*, and *PIK3R1* and the downstream-acting genes *ATF*, *CHUK*, and *MDM2* indicated that they were upregulated (Fig. 6d–i). The sarcomere maturation genes *MYH10* and *MYLK* also increased (Fig. 6j, k); however, the members of the PI3K-Akt signalling pathway *AKT*, *PIK3CA*, and *PIK3R1* were unchanged on days 7, 9, and 11 (Supplementary Fig. S6). These results indicate that PYGO2 induces the formation and subsequent differentiation of hUC-MSCs into cardiomyocytes through the PI3K-Akt signalling pathway during the middle-late stages.

## Discussion

MSCs have beneficial therapeutic effects on heart disease by differentiating and replacing damaged cardiac tissue cells. Therefore, they represent a promising treatment strategy, although the underlying molecular mechanism effects are not fully understood. Canonical Wnt signalling promotes the differentiation of MSCs into cardiomyocytes, although the role of its key member PYGO2, which uses the same mechanism to promote hUC-MSCs differentiation into cardiomyocytes, is unclear. Here, we used hUC-MSCs to identify the molecular mechanism through which PYGO2 regulates the differentiation into and formation of cardiomyocytes using a system that represents in vivo physiological conditions. Our results provide compelling evidence that PYGO2 contributes to canonical Wnt signalling in the early stages to promote the differentiation into mesodermal-like cells and cardiac progenitors from hUC-MSCs. However, we unexpectedly found that PYGO2 was not associated with certain signalling pathways, such as the NOTCH and BMP pathways, but can regulate the PI3K-Akt signalling to promote the differentiation of hUC-MSCs into cardiomyocyte-like cells.

Stem cells are types of cells with differentiation potential, and include induced pluripotent stem cells (iPSCs) (Protze et al. 2019), mesenchymal stem cells (Colicchia et al. 2019), and skeletal myoblasts (Rikhtegar et al. 2019). iPSCs, which represent an early, pre-gastrulation stage, mediate the key stages of heart development in the early embryo. Adding exogenous adaptive factors at different time-points accelerates the differentiation of iPSCs into cardiomyocytes. For example, once differentiation commences, adding activators of the canonical Wnt, nodal, and BMP signalling pathways accelerates mesodermal formation and promotes cardiomyocyte differentiation (Colicchia et al. 2019).

MSCs are relatively easy to isolate and can improve cardiac function in patients with heart failure (Fisher et al. 2014); of all MSCs, hUC-MSCs are one of the three most widely used. Certain external stimuli induce hUC-MSCs to differentiate into cells representing the three germ layers, regulate the immune response, and accumulate in damaged tissues or inflamed areas to promote tissue repair (Colicchia et al. 2019). Moreover, hUC-MSCs possess significant pro-angiogenic and anti-fibrotic properties (Colicchia et al. 2019). Similarly, changing the culture conditions of hUC-MSCs also promotes their differentiation into cardiomyocyte-like cells (Sun et al. 2020). During the differentiation of stem cells into cardiomyocytes in vitro, canonical Wnt signalling induces the differentiation into cardiomyocytes.

Human iPSCs (hiPSCs), treated with exogenous canonical Wnt signalling activators once differentiation commences, increase mesoderm formation (Lian et al. 2012). In mouse embryonic stem cells (mESCs), early induction of β-catenin expression promotes the proliferation of mESCs to favour early stem cell commitment towards the mesoderm. Continuous induction of β-catenin expression promotes the commitment of mESCs to undergo endodermal differentiation (Pedone et al. 2022). In hiPSCs, increased canonical Wnt signalling activity accelerates the differentiation of hiPSC into the cardiac mesoderm (Buikema et al. 2020).

Here, we demonstrated that hUC-MSCs (Vector group) lack detectable cTnT-positive cardiomyocyte-like cells on day 14 and approximately 5% of such cells appeared until day 21. These results indicate that hUC-MSCs primarily transform into mesodermal cells and cardiac progenitor cells before day 14 and that some cardiomyocyte-like cells begin forming after day 14. In the PYGO2 group, the expression of the mesodermal markers *T* (*Brachyury*) and *MIXL1* was significantly increased on day 7 and the levels of the myocardial progenitor markers *MESP1*, *GATA4*, *NKX2.5*, and *TBX5* were significantly upregulated on day 9 (Figs. 1 and 2), indicating that PYGO2 induces hUC-MSCs to initiate the formation of mesodermal-like cells and cardiac progenitor cells in advance. Following treatment with XAV939, an inhibitor of canonical Wnt signalling, the expression of genes encoding PYGO2 and β-catenin, and the cardiac progenitor markers *GATA4* and *NKX2.5* decreased (Fig. 3). These results indicate that PYGO2 promotes the formation of cardiac mesodermal-like cells and cardiac progenitor cells in a canonical Wnt signalling pathway-independent manner. This is similar to the findings that β-catenin overexpression promotes stem cells to commit early toward the mesodermal lineage.

Canonical Wnt signalling plays an important temporal role in heart development. During the developmental stages of the three germ layers, canonical Wnt signalling induces the expression of mesodermal transcription factors and promotes the formation of cardiac progenitor cells from mesodermal cells, thereby promoting the expression of specific cardiac transcription factors (Burridge et al. 2015; Foulquier et al. 2018). After cardiac field formation, canonical Wnt signalling is inhibited by the first and second heart fields to maintain gene expression and promote cardiomyocyte differentiation and maturation (Burridge et al. 2015; Foley and Mercola 2005). For example, in mouse models, the absence of β-catenin or Wnt3a results in abnormal cardiac mesoderm formation (Huelsken et al. 2000; Kwon et al. 2007; Liu et al. 1999).

Inhibitors of canonical Wnt signalling, such as Frizzled and Dkk-1, induce cardiac gene expression in the posterior lateral plate mesoderm (Marvin et al. 2001). Our previous study showed that PYGO, which does not regulate the formation of adult cardiac valves, functions through canonical Wnt signalling (Lin et al. 2021; Tang et al. 2013). While investigating hUC-MSCs differentiation and cardiomyocyte-like cell formation, we adopted a different approach not used in other studies (Joshi et al. 2018; Pham et al. 2016; Ruan et al. 2016). We did not add any other reagents to cells ectopically overexpressing *PYGO2* to promote the differentiation of hUC-MSCs into cardiomyocytes under conditions that faithfully represented those found in vivo. However, in the XAV939 experiment, it should be noted that in overexpressed PYGO2 group, after adding DMSO, β-catenin and its target genes, such as *Cyclin D1*, *TCF*, and *ACTA1*, did not change at mRNA level (Fig. 3h, i). We have repeated the experiment several times, and the results were same. The affect factors are unknown.

Unexpectedly, we found that constitutive PYGO2 overexpression did not affect the expression of canonical Wnt signalling genes in the middle–late stages of hUC-MSCs differentiation (Fig. 5a). This indicates that PYGO2 does not regulate this pathway to promote the differentiation of hUC-MSCs into cardiomyocytes during this stage. Furthermore, PYGO2 did not regulate certain signalling events that regulate cardiomyocyte differentiation, such as BMP signalling or NOTCH signalling that promotes the differentiation of cardiomyocytes during this stage (Fig. 5b–e). PYGO2 overexpression, which generated a cell population consisting of approximately 3% positive cardiomyocytes on day 14, was not detected in the control group (Fig. 2c–f). Therefore, we selected cells at this stage for RNA-seq and bioinformatic enrichment analysis. An analysis of enriched KEGG pathways identified the PI3K-Akt signalling pathway (Fig. 6b), which is related to the formation of cardiomyocytes at this stage. This result indicates that PYGO2 significantly activates the expression of PI3K-Akt signalling–related genes during differentiation of hUC-MSCs into cardiomyocytes (Fig. 6d). Thus, our unique approach indicates a novel function of PYGO2, in which PYGO2 activates the PI3K-Akt signalling pathway to promote the differentiation and formation of hUC-MSCs into cardiomyocytes during the middle–late stages.

The PI3K-Akt signalling pathway acts as a key regulator of various cellular processes including cardiomyocyte maturation. For example, during the early stage of cardiac tissue development, the expression of *AKT* gene is inhibited and that of the SHF gene is increased, which promotes the specialization of myocardial progenitors (Bisson et al. 2015). During the later stage, the expression of *AKT* increases, whereas that of the SHF genes are inhibited. In addition, the expression of myocardial cell maturation makers are increased, indicating the maturation of myocardial cells.

The PI3K-Akt signalling pathway is upregulated during the canonical Wnt signalling-independent induction of cardiomyocyte maturation (Buikema et al. 2020), which is consistent with our current results. The RNA-seq data show that another pathway, included in the KEGG pathway TOP 10, regulates the actin cytoskeleton (Fig. 6b). This indicates further indicating that PYGO2 regulates cardiomyocyte maturation through PI3K-Akt during the middle and late stages, although numerous genes that regulate cardiomyocyte maturation were not activated during this stage.

PYGO2 binds to DNA-binding proteins (ChiLS) to regulate transcriptional activity by modifying lysine residue 4 of the histone 3 tail (H3K4) bound to chromosomes in an epigenetic-related manner (Shi et al. 2020b). Moreover, in a mouse model of testis development, the histone acetyl transferase Gcn5 was recruited to acetylate histones to induce the gene expression required between the stages of sperm cell differentiation (Cantù et al. 2013). Pygo2 directly binds to the promoter region of *Pax6* to regulate its expression, which affects the development of the mouse lens (Cantù et al. 2014). Furthermore, in human breast cancer-derived MBA-MB231 cells, PYGO2 interacted with H3K4me2/3 to regulate mammosphere formation (Chen et al. 2010). Therefore, PYGO2 may act as a histone co-factor to regulate the expression of genes associated with the PI3K-Akt signalling pathway, thus influencing the continuous differentiation of hUC-MSCs into cardiomyocyte-like cells. However, the specific molecular mechanisms remain to be identified.

## Conclusion

In summary, we demonstrated that PYGO2 regulates the biphasic differentiation of hUC-MSCs into cardiomyocytes. PYGO2 relies on canonical Wnt signalling in the early stage to promote the formation of mesodermal-like cells and the differentiation of cardiac progenitors from hUC-MSCs during early-stage. PYGO2 regulates the PI3K-Akt signalling pathway to promote the differentiation of hUC-MSCs into cardiomyocyte-like cells during middle-late stage. Further studies of this mechanism will likely have important implications for the development of clinical applications for cell therapy using hUC-MSC-derived cardiomyocytes.

## Supplementary Information

Below is the link to the electronic supplementary material.

Supplementary file1 (DOCX 3545 KB)

## Declarations

The hUC-MSCs were isolated from umbilical cords, which were obtained from healthy infants delivered by caesarean section at the Third Xiangya Hospital of Central South University in December 2019 and the Ethics Committee of this hospital approved this study (no. 20042). This study was approved by the Ethics Review Board of the Guangdong Provincial People’s Hospital (KY-Z-2021–609-01).
