# Supplementary material for: Novel biphasic mechanism of the canonical Wnt signalling component PYGO2 promotes cardiomyocyte differentiation from hUC-MSCs
Source: Cell Tissue Res. 2023 May 26;393(1):163–79. doi: 10.1007/s00441-023-03774-6 (PMC10313541; doi:10.1007/s00441-023-03774-6)
Supplement: Supplementary file 1 — Supplementary file1 (DOCX 3545 KB) [file 441_2023_3774_MOESM1_ESM.docx]

# Novel biphasic mechanism of canonical Wnt signalling component PYGO2 promotes cardiomyocyte differentiation from hUC-MSCs

Yan Shi^1^,^3#^, Bin Qin^2#^, Xiongwei Fan^1#^, Yongqing Li^2#^, Yuequn Wang^2#^, Wuzhou Yuan^2^, Zhigang Jiang^2^, Ping Zhu^1,4^, Jimei Chen^1^, Yu Chen^1,4^, Fang Li^2^, Yongqi Wan^2,^*, Xiushan Wu^2,4^*, Jian Zhuang^1,3^*

^1^Guangdong Cardiovascular Institute, Guangdong Provincial People's Hospital, Guangdong Academy of Medical Sciences, Guangzhou, Guangdong 510100, China

^2^The Center for Heart Development, State Key Laboratory of Development Biology of Freshwater Fish, College of Life Sciences, Hunan Normal University, Changsha, Hunan 410081, China

^3^Laboratory of Artificial Intelligence and 3D Technologies for Cardiovascular Diseases, Guangdong Provincial Key Laboratory of South China Structural Heart Disease, Guangdong Provincial People’s Hospital, Guangdong Academy of Medical Sciences, Guangzhou, 510080, P.R. China

^4^Guangdong Provincial Key Laboratory of Pathogenesis, Targeted Prevention and Treatment of Heart Disease, Guangzhou, Guangdong 510080, P.R. China

^#^These authors contributed equally to this work

*To whom correspondence should be addressed

Xiushan Wu, PhD

Director and professor

The Center for Heart Development

Hunan Normal University

Changsha, Hunan 410081, China

TeL: 0086-0731-88872780

Email: [xiushanwu2003@aliyun.com](mailto:xiushanwu2003@aliyun.com)

# Supplementary Figures Legends


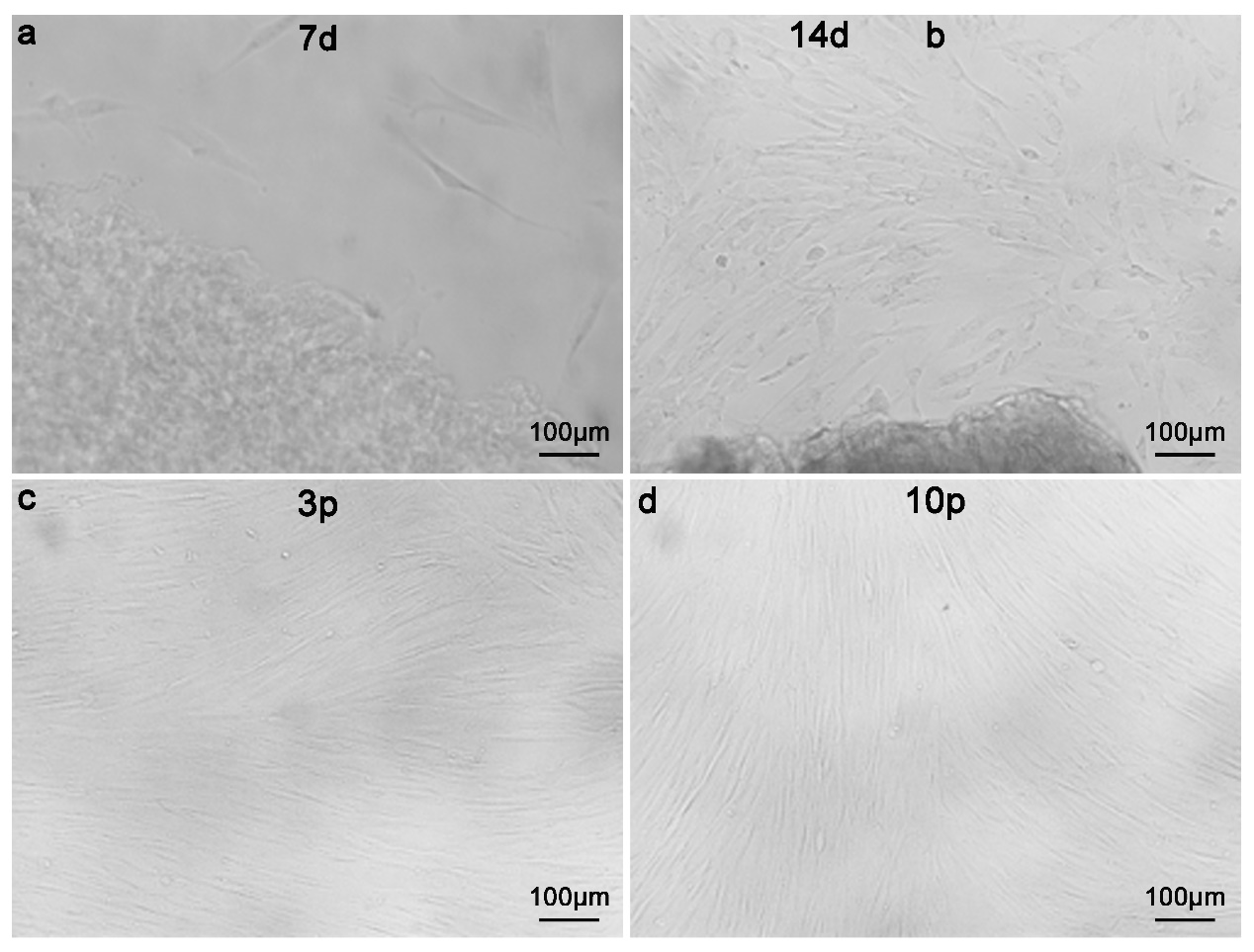


## Supplementary Figure S1 Morphology of hUC-MSCs at different days and passages

**a-b** Primary HUC-MSCs on days 7 and 14 of tissue-block culture (×200, and ×100, respectively); **c-d** Passages of HUC-MSCs for 3 and 10 generations, respectively (×100). d, days, days after tissue isolation; p, passages, passage number.


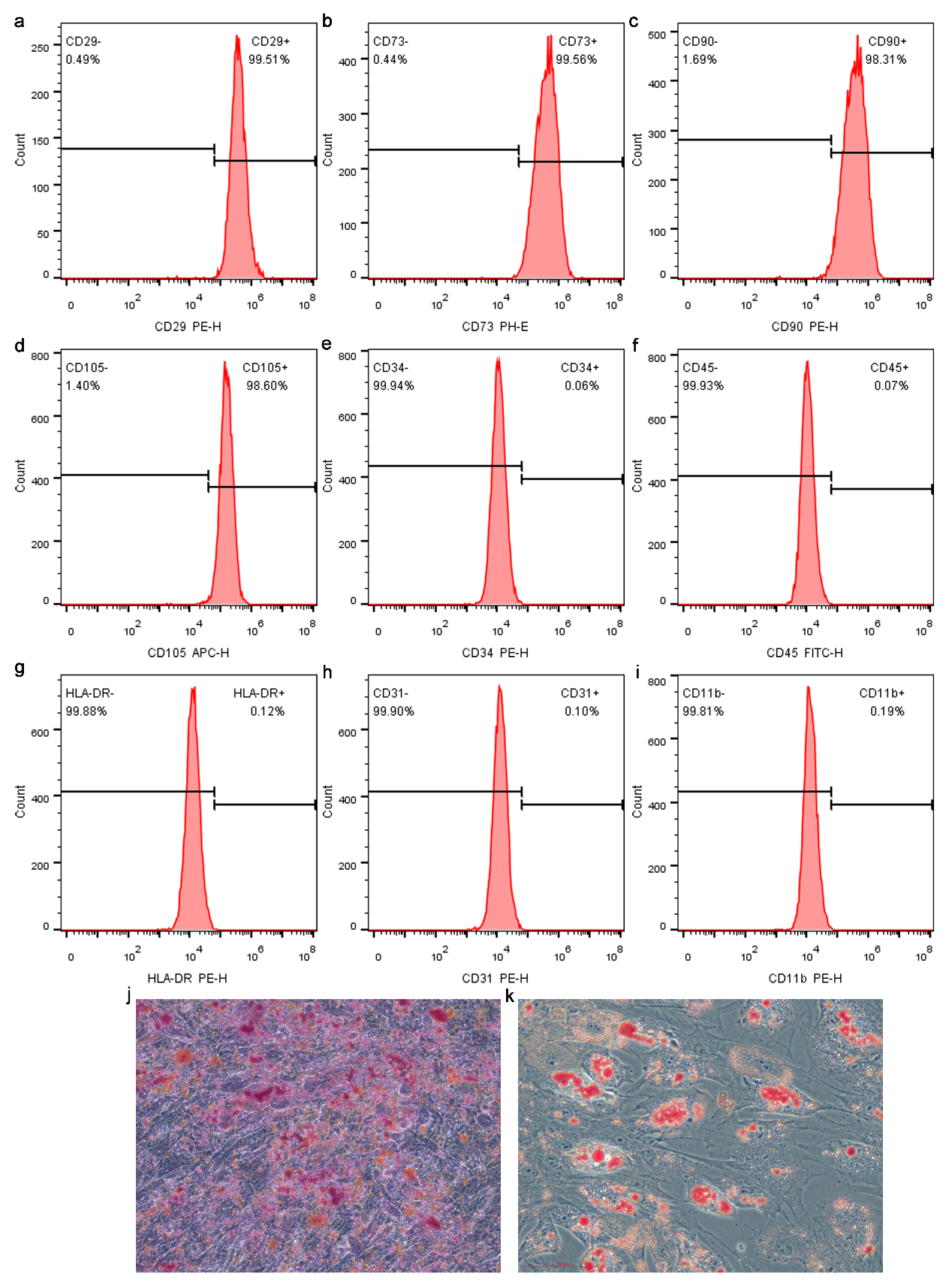


## Supplementary Figure S2 Analysis of the differentiation potential of theP3 generation HUC-MSCs

**a-i** Flow cytometry analysis of the immunophenotype of P3 generation HUC-MSCs. **j-k** Adipogenic differentiation of HUC-MSCs (Oil Red O staining, ×400); B, Osteogenic differentiation of HUC-MSCs (Alizarin S staining, ×200).


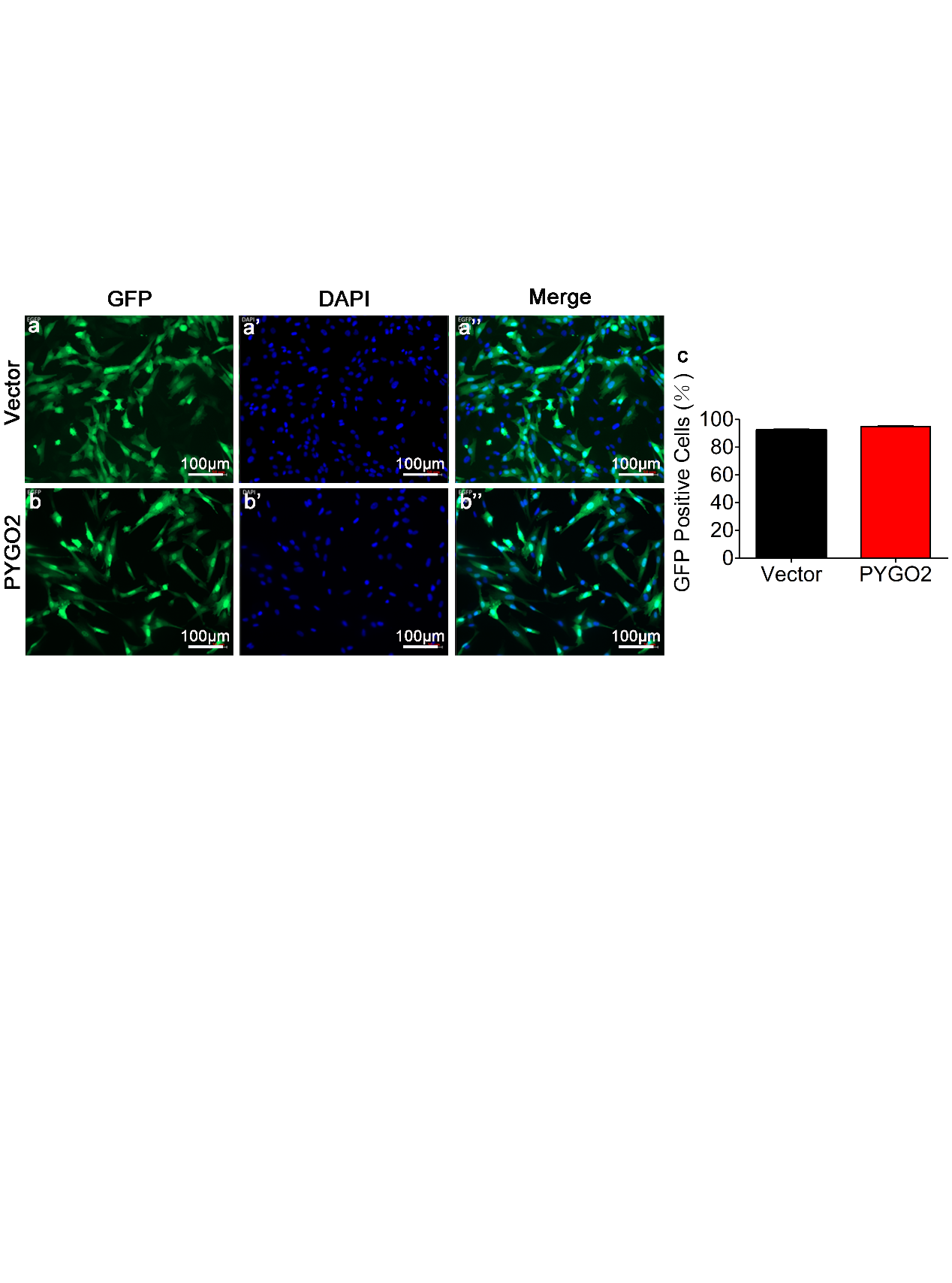


## Supplementary Figure S3 Establishment of PYGO2 stable system

**a-b’’** After puromycin treatment, the proportion of GFP-positive cells was determind. Scar bar = 50μm. **c** Quantitative plot of a and b, percentage of GFP-positive cells (GFP) (DAPI) (GFP/DAPI×100%). The error bars indicate the mean and SD.


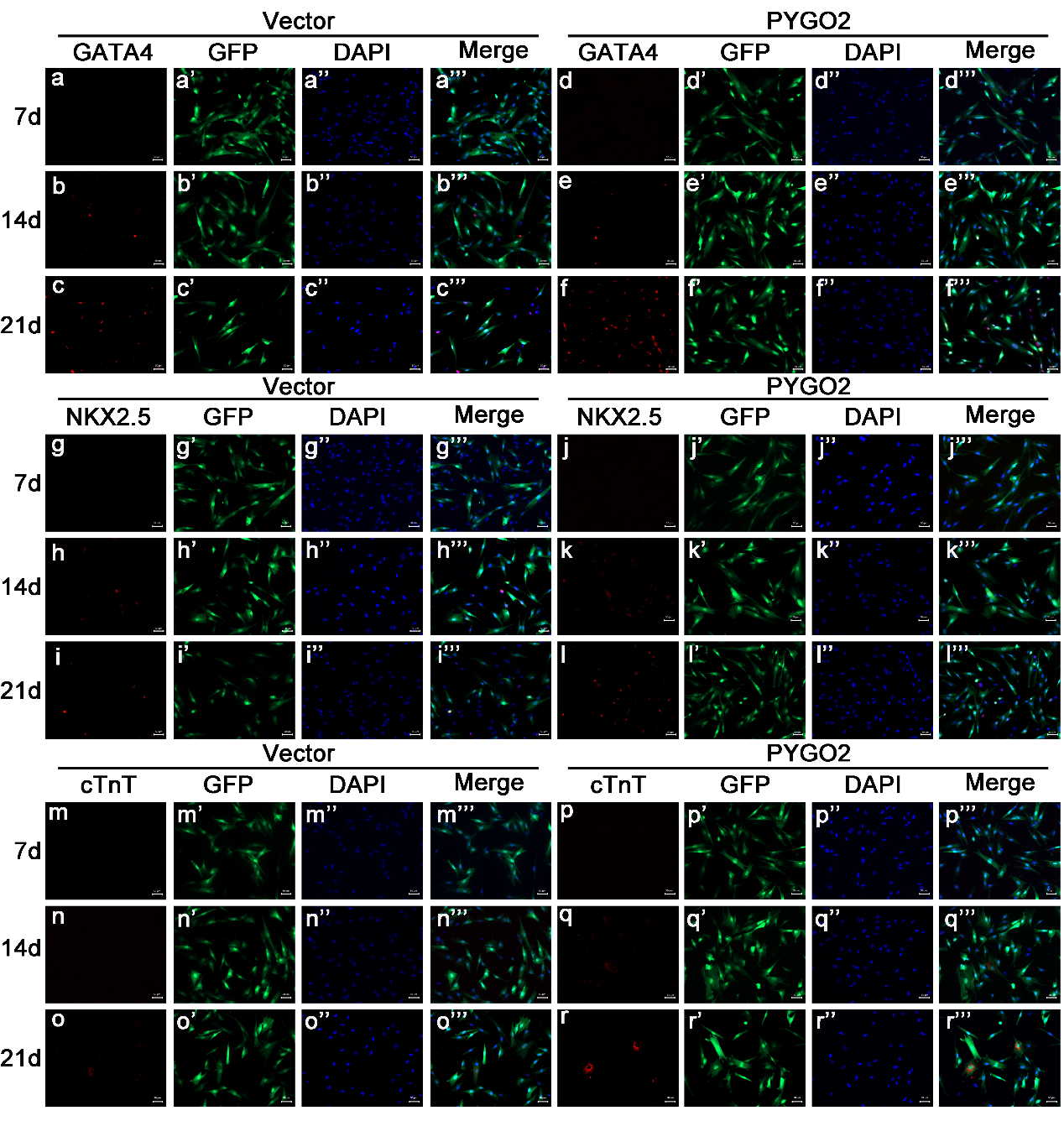


## Supplementary Figure S4 Immunofluorescence analysis of the temporal expression of NKX2.5, GATA4 and cTnT

**a-f** Immunofluorescence detection of and GATA4-positive cells at different days. **g-l** Immunofluorescence detection of and NKX2.5-positive cells at different days. **m-r** Immunofluorescence detection of and cTnT-positive cells at different days. d, days after the establishment of stable PYGO2 overexpression. Scar bar: 50μm.


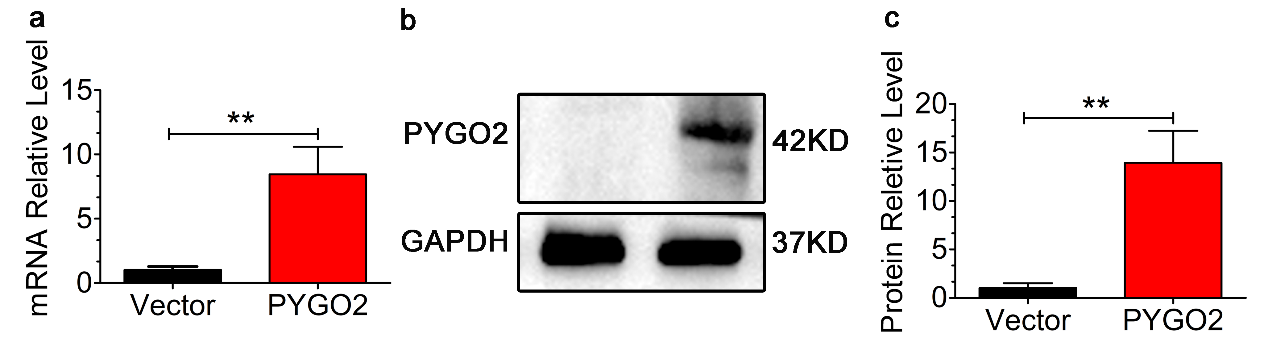


## Supplementary Figure S5 Establishment of PYGO2 stable system in 293T cell line

**a** The expression of PYGO2 was detected on day 4 using qRT-PCR. **b** The expression of PYGO2 was detected on day 4 using western blot. **c** Quantitative plot of b.

Vector, the group infected with empty vector, was used as control. PYGO2, the group that overexpressed PYGO2; **, *p* < 0.01; The error bars indicate the mean and SD.


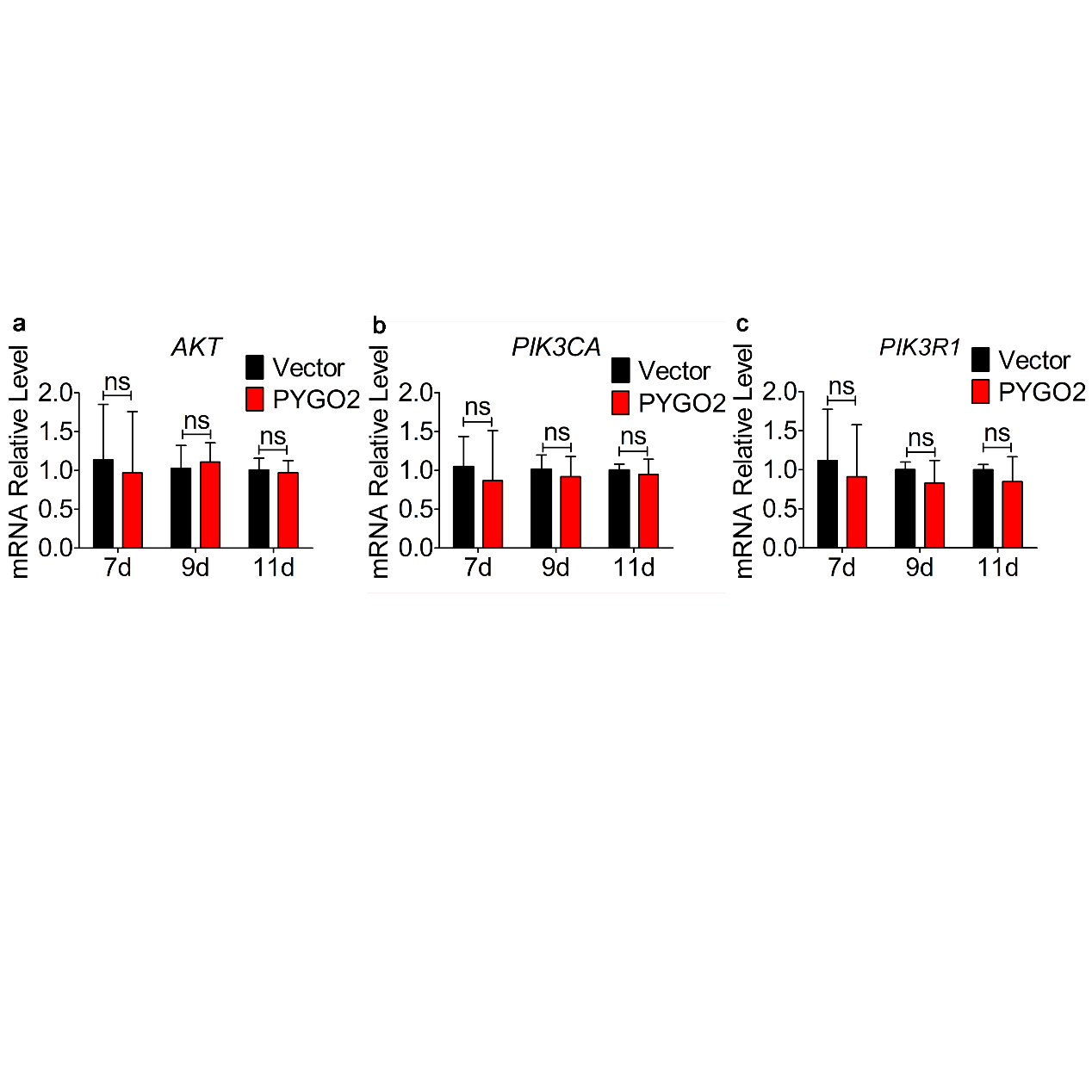


## Supplementary Figure S6 The expression of PI3K/AKT pathway members in the early stages

**a, b** and **c** qRT-PCR analysis detected the expression of *AKT*, *PIK3CA* and *PIK3R1* on day 7, 9 and 11. Vector, the group infected with empty vector, served as the control; PYGO2, the group that overexpressed PYGO2; d, days; ns, *p* > 0.05; The error bars show the mean and SD.

# Supplementary Table S1

Supplementary Table S1 qRT-PCR Primers used in this article

| *Gene* | Left primer | Right primer |
| --- | --- | --- |
| *PYGO2* | TCTCTGTCCCAACGATTTGCT | GCAGGTGGATTCAAGGGCT |
| *T* | CAAGCTCACCAACAAGCTCA | TGAACTGGGTCTCAGGGAAG |
| *MIXL1* | GTTCCAGGAGCACAGTGGTT | AGCTGCTGGAGCTCGTCTT |
| *MESP1* | TGACAAGGGACAACTGACGC | CTAGCCCTATGGGTCCCTCC |
| *GATA4* | GTGTCCCAGACGTTCTCAGTC | GGGAGACGCATAGCCTTGT |
| *NKX2.5* | CTTCAAGCCAGAGGCCTACG | TCAGGCTTTCTTTTCGGCTCTAG |
| *TBX5* | CTGTGGCTAAAATTCCACGAAGT | GTGATCGTCGGCAGGTACAAT |
| *MEF2C* | AGATTACGAGGATTATGGATGAAC | CTTGTTGGTGCTGTTGAAGATGA |
| *MYH6* | GAGTCGGTGAAGGGCATGAG | TTTGCTTGGCACCAATGTCAC |
| *SOX2* | TACAGCATGTCCTACTCGCAG | TGGAGTGGGAGGAAGAGGTAAC |
| *OCT4* | TCGAGAAGGATGTGGTCCGA | GAAGTGAGGGCTCCCATAGC |
| *β-catenin* | GCTGAAGGTGCTATCTGTCTGC | CCTTCCATCCCTTCCTGTTTAG |
| *WNT3* | AGGGCACCTCCACCATTTG | GACACTAACACGCCGAAGTCA |
| *ACTC1* | TCTTCCAGCCCTCCTTCATTG | AGCCAGAGCAGTGATTTCCTTC |
| *TCF* | ATTAGCGAGAGGGTCTGAGC | AGTTTTGCACACGGTCAGTC |
| *Cyclin D1* | ATGCCAACCTCCTCAACGAC | CTCCTCGCACTTCTGTTCCTC |
| *GAPDH* | GGAAGCTTGTCATCAATGGAAATC | TGATGACCCTTTTGGCTCCC |
| *SMAD4* | CCATTTCCAATCATCCTGCT | ACCTTTGCCTATGTGCAACC |
| *BMP4* | TGATACCTGAGACGGGGAAG | ATGTTCTTCGTGGTGGAAGC |
| *NOTCH3* | TGTGGACGAGTGCTCTATCG | AATGTCCACCTCGCAATAGG |
| *HES1* | TGAAAGTCTGAGCCAGCTGA | GTCACCTCGTTCATGCACTC |
| *ACTA2* | gtcacccacaatgtccccat | tccgatggtgatcacttgcc |
| *TPM1* | ggcaccgaagatgaactgga | tgggcacgatccaactcttc |
| *MYLK* | ggaaatcctcaacagggtca | ctagcagcacttccctccac |
| *MYH10* | acctgaaattacggcactgg | cttccaccttcgtctgcttc |
| *CHUK* | tatgaagggccatttgcttc | actgcttcagcccacacttt |
| *MDM2* | cagcttcggaacaagagacc | gtccgatgattcctgctgat |
| *ATF2* | ccccaacaccaacaagattc | tgctggacgaacaatagctg |
| *PIK3CA*  *PIK3R1* | gtcaatcggtgactgtgtgg  cgctttcaaacgctatctcc | tccatcgtctttcaccatga  agagctggctgctgagaatc |
